# Preliminary study of Yinhuapinggan granule against H1N1 influenza virus infection in mice through inhibition of apoptosis

**DOI:** 10.1080/13880209.2020.1818792

**Published:** 2020-09-23

**Authors:** Hai-xia Du, Hui-fen Zhou, Jie-hong Yang, Yi-yu Lu, Yu He, Hai-tong Wan

**Affiliations:** a College of Basic Medical Science, Zhejiang Chinese Medical University, Hangzhou, China;; b College of Life Science, Zhejiang Chinese Medical University, Hangzhou, China;; c Institute of Microbiology, Zhejiang Center for Disease Control and Prevention, Hangzhou, China;; d College of Pharmaceutical Science, Zhejiang Chinese Medical University, Hangzhou, China

**Keywords:** Traditional Chinese medicine, apoptosis signalling pathway

## Abstract

**Context:**

Yinhuapinggan granule (YHPG) is frequently used for treating fever, cough, and viral pneumonia in traditional Chinese medicine.

**Objective:**

This study investigated the antiviral effects of YHPG in H1N1 influenza virus (IFV)-infected mice and its possible mechanism.

**Materials and methods:**

ICR mice were intranasally infected with 10 LD_50_ viral dose of IFV and then oral administration of YHPG (6, 12, and 18 g/kg) or oseltamivir (positive control) once a day for 2 or 4 consecutive days, six mice in each group. The lung, spleen and thymus indexes of IFV-infected mice, the expression of viral loads and pathological changes in lung tissues were performed to evaluate the antiviral effects of YHPG. Real-time PCR, immunohistochemistry and western blot assays were used to determine the expression of Bax, Bcl-2 and caspase-3.

**Results:**

LD_50_ in mice was 10^−3.5^/0.02 mL. YHPG (6, 12, and 18 g/kg) dose-dependently decreased the lung index and viral load; the inhibition ratio of lung index was 5.31, 18.22, and 34.06%, respectively. Further detection revealed that YHPG (12 and 18 g/kg) significantly attenuated lung pathological changes, and increased the spleen and thymus indexes. Moreover, YHPG significantly down-regulated the mRNA and protein expression of Bax and caspase-3 in lung tissues of mice infected with IFV, and up-regulated the expression of Bcl-2.

**Conclusions:**

YHPG has significant antiviral effects in IFV-infected mice, partially by inhibiting influenza virus replication and regulating the occurrence of apoptosis induced by influenza virus infection, suggesting that YHPG may be a promising antiviral agent with potential clinical application prospects.

## Introduction

Influenza viruses are major pathogens in humans and animals, and with their ability to cause seasonal epidemic and occasional pandemics in the human population that are responsible for significant morbidity and high mortality, they pose a considerable burden on the global health care systems (Paterson & Fodor 2012). In addition, viral diseases cause many serious agricultural diseases throughout the modern era of veterinary medicine, and pose serious threats to the food animal production, which then have negative impacts on human health (Arzt et al. 2010). The recent emergence of the avian influenza A virus (H7N9), the 2009 pandemic influenza A virus (H1N1pdm09), the avian influenza A virus (H5N1), and severe acute respiratory syndrome-related coronaviruses (SARSr-CoVs), are important causative agents of viral pneumonia and have a significant impact on the national economy (Shinya et al. 2012; Simonsen et al. 2013; Kang et al. 2015; Zhou et al. 2020). Currently, the existing antiviral medications approved by the United States (US) Food and Drug Administration (FDA) for management of influenza virus infections mainly include M2 ion channel inhibitors (e.g., amantadine and rimantadine), neuraminidase inhibitors (e.g., zanamivir and oseltamivir) and RNA polymerase inhibitors (e.g., ribavirin) (Okomo-Adhiambo et al. 2013; Fatima et al. 2016). Despite their availability, possible side-effects, drug resistance, and compliance are the potential problems of these drugs (Okomo-Adhiambo et al. 2013; Wan et al. 2013). Furthermore, the influenza A virus is susceptible to antigenic drift or antigen transformation and recombination, resulting in the emergence of new subtypes and lack of immunity in most populations (Wan et al. 2013). For the influenza virus, theses pandemic strains that have been extensively altered by antigenic variation have prevented the production of universal vaccines (Sherbany et al. 2014). In addition, the high cost of effective and convenient therapeutic agents has also limited their clinical application. Therefore, there is still an urgent need to develop new anti-influenza drugs.

Apoptotic cell death is essential for embryonic development, tissue homeostasis, and a well-functioning immune system, with aberrant apoptosis contributing to numerous disease conditions (Dewson & Kluck 2010), and apoptosis is a genetically controlled process distinct from necrosis. It is characterised by chromatin condensation, DNA fragmentation, membrane vesicles, cell shrinkage, and finally formation of apoptotic bodies. Apoptosis can be triggered by a variety of physiological and pathological stimuli, and it widely exists in several immunosuppressive diseases of humans and animals (Puerto et al. 2010). Influenza A virus infection has been shown to induce apoptosis in a variety of cell types both *in vitro* and *in vivo*, and the mechanisms of apoptosis are highly complex, involving an energy-dependent cascade of molecular events (Herold et al. 2012; Liu et al. 2014). Viral infection and viral replication activate multiple cell signalling pathways. Influenza A virus infection induces typical apoptosis involving two main apoptotic pathways: the extrinsic or DR pathway and the intrinsic or mitochondrial pathway, which may be linked to each other (Yang et al. 2012). Influenza A virus generally causes caspase-dependent apoptosis based on caspase-3 activation, which participates in a tightly regulated proteolytic cascade (Wong 2011). The BCL-2 family of proteins regulates apoptotic cell death in many cell types, and the balance between anti-apoptotic and pro-apoptotic family members determines cell death or survival by controlling apoptosis (Tuzlak et al. 2017). However, there are few studies about the possible apoptotic molecular mechanisms associated with pathogenesis of influenza A virus infection *in vivo*. For the above reasons, the regulation of apoptosis has become an important target for anti-influenza treatment.

The use of herbal medicine has been accepted in many countries including areas where healthcare systems have been improved, and medicinal plants as complementary therapies and as preventive medicine are becoming more and more popular in modern society (Mehrbod et al. 2018). Traditional Chinese medicine, due to its minimal side effects, multi-target and multi-channel characteristics, has become the popular choice in the treatment and prevention of influenza virus infection. Ma-Huang-Tang (MHT), the prestigious and typical formula first recorded in Zhang Zhongjing’s Treatise on Febrile Disease, is considered to be the classical prescription for virus infection and has been traditionally used to treat serious infectious diseases, including cough, pneumonia, and viral diseases (Saita et al. 2011; Nagai et al. 2014). Yinhuapinggan granule (YHPG), is a kind of MHT modified prescription based on Professor Wan Haitong’s clinical experience, which contains Radix Puerariae Lobatae (the radix of *Pueraria lobata* (Willd.) Ohwi [Lamiaceae]), Flos Lonicerae Japonicae (the flower bud of *Lonicera japonica* Thunb. [Caprifoliaceae]), Polygoni Cuspidati Rhizoma (the root and rhizome of *Polygonum cuspidatum* Sieb.et Zucc. [Polygonaceae]), Ephedrae Herba (the aerial part of *Ephedra sinica* Stapf. [Ephedraceae]), Armeniacae Semen Amarum (the fruit of *Prunus armeniaca* L. [Rosaceae]), Glycyrrhizae Radix (the root and stolon of *Glycyrrhiza uralensis* Fisch. ex DC [Leguminosae]). According to the basic composition principle of Chinese traditional prescriptions - ‘monarch, minister, assistant, and guide’, it has been reported that several components of YHPG and their constituents play antiviral and anti-apoptosis effects against several viruses. The flower buds of *Lonicera japonica* have the activity of inhibiting Coxsackie virus B3 and influenza virus *in vitro* (Kashiwada et al. 2013; Yu et al. 2015). Ephedrae herba is a Japanese herbal medicine, which has a certain effect on the infection of influenza A virus infection *in vivo* and *in vitro* (Mantani et al. 1999; Kubo and Nishimura 2007). Radix Puerariae Lobatae is proven to have antiviral activity against HRSV-induced plaque formation in airway mucosa mainly by inhibiting viral attachment and internalisation (Lin et al. 2013). Polygoni Cuspidati Rhizoma is an effective agent for antiviral, anti-inflammation, anti-apoptosis and inhibiting the influenza virus replication (Peng et al. 2013; Lin et al. 2015). And the last, glycyrrhizin interfered with the replication of influenza virus, inhibited the expression of virus-induced pro-inflammatory cytokines, interfered with H5N1-induced apoptosis and reduced virus uptake *in vitro* (Wolkerstorfer et al. 2009; Michaelis et al. 2011). In previous research, YHPG has been shown to inhibit the reproduction of H1N1 influenza virus in embryonic eggs as a mixture of six herbs in different proportions (Peng et al. 2005). In addition, YHPG has obvious protective effects on IFV-infected mice associated with alleviation of lung injury, regulation of cytokine production via NF-κB p65 activation and inhibition of TLR4-MyD88-TRAF signalling pathway *in vivo* (Peng et al. 2016a, 2016b). *In vitro*, YHPG also has certain antiviral effects in IFV-infected RAW264.7 cells, which might be associated with regulation of inflammatory cytokines production, inhibition of TLR7-MyD88-IRF7 signalling pathway, activation of NF-κB p65, and modulation of the protein expressions of key effectors in the Type I IFN and PRRs signalling pathways (Du et al. 2017, 2018). Clinically, the Phase II and Phase III clinical trials of YHPG have been proved to treat infectious disease with good curative effect. But to date, the possible antiviral mechanism of YHPG against IFV-induced lung injury in apoptosis respect remains unclear. In this study, we evaluated the antiviral effects of YHPG against H1N1 influenza virus infection in the ICR mouse model, and mainly investigated its possible mechanism focus to key targets Bax, Bcl-2 and caspase-3 in apoptosis signalling pathway.

## Materials and methods

### Material and reagents

YHPG granules (batch number: 1606020) were supplied by Hangzhou Huadong Medicine Group Kangrun Pharmaceutical Co., Ltd. (Hangzhou, China), and were identified by Prof. Shengwu Huang, College of Pharmaceutical Science, Zhejiang Chinese Medical University (Hangzhou, China). According to the traditional dose ratio of 4 (Radix Puerariae Lobatae): 4 (Flos Lonicerae Japonicae): 4 (Polygoni Cuspidati Rhizoma): 2 (Ephedrae Herba): 2 (Armeniacae Semen Amarum): 1 (Glycyrrhizae Radix) (Supplementary Table 1), YHPG granules were prepared by water-extraction and ethanol-precipitation, and the quality control of YHPG was also performed by HPLC, as reported in our previous research. HPLC analysis was carried out at 210 nm for the determination of eight active components in YHPG, containing L-ephedrine (0.73 mg/g), D-pseudoephedrine (0.407 mg/g), chlorogenic acid (1.92 mg/g), amygdalin (6.37 mg/g), puerarin (4.35 mg/g), polydatin (1.79 mg/g), glycyrrhizic acid (0.692 mg/g) and emodin (0.329 mg/g), respectively (Supplementary Figure 1) (Peng et al. 2016a, 2016b).

Oseltamivir phosphate capsules (Specification: 75 mg, batch number: SH0037) were obtained from Roche Pharmaceutical Co., Ltd. (Shanghai, China) (import packing). Rabbit-anti-mouse polyclonal antibody for Bax, Bcl-2 and caspase-3 were purchased from Santa Cruz Biotechnology (CA, USA).

### Virus strain

Mouse-adapted influenza A/PR/8/34 virus (H1N1 subtype), a gift from the Zhejiang Provincial Centre for Disease Control and Prevention (Hangzhou, China), was stored in aliquots at −80 °C. The influenza virus caused viral pneumonia model in mice. Mice with influenza viral pneumonia model were induced by intranasal inoculation with IFV under ether anaesthesia, and 50% lethal dose (LD_50_) of IFV in mice determined by Reed-Muench method was calculated to be 10^−3.5^/0.02 mL.

### Animal groups and experimental design

Male Institute of Cancer Research (ICR) mice weighing 18–22 g were supplied by the Central Animal Facility of Zhejiang Academy of Medical Sciences [SYXK (Zhe) 2016-0001]. Mice were housed on the standard laboratory room with free access to chow and tap water, at the controlled temperature of 22 ± 2 °C, and under a 12 h light/darkness cycle. All animal experimental procedures were performed in compliance with the guide for the care and use of laboratory animals, published by the National Institutes of Health (USA, No.80-23, revised in 1996). The study was approved by the Animal Experiment Protection and Use Committee of Animal Experimental Centre in Zhejiang Chinese Medical University (Ethic approval code: No. ZSLL-2015-126) and performed according to its guidelines.

The mice were intranasally challenged with 10 LD_50_ (50% lethal dose) H1N1 in a volume of 20 μL under mild anaesthesia with ether according to our previous research (Wu et al. 2016), and divided randomly into influenza virus infection control group (IFV-C), oseltamivir-treated group (positive control, oseltamivir; 21.63 mg/kg), YHPG-treated group at doses of 6, 12 and 18 g/kg (six mice in per group on each time point). The other mice were intranasally infected with equal amount of physiological saline as the normal control group (Normal-C). At 2 h after infection, YHPG and oseltamivir were administrated by oral gavage to the mice of treatment groups once daily for 2 or 4 d, respectively. The volume of oral administration was 0.2 mL/10 g mice. The mice in Normal-C and IFV-C were given saline solution at the same time interval. During the whole experiment period, all the mice could freely get chow and tap water. The drug dose setting was based on the YHPG daily dose of the adult 60 kg weight, which was 42.5 g per day. The low dose of YHPG (6 g/kg) in the mouse experiment was equivalent to the human dose clinical practice. Considering that the concentration of YHPG was 30 g/kg previously, and the dose was slightly higher. We chose the maximum dose 18 g/kg here, which was three times the human clinical equivalent dose.

### Lung index, spleen index and thymus index

On the third and fifth days after influenza virus infection, six mice in each group were randomly euthanized by ether anaesthesia and bloodletting, and lung tissues, spleen tissues and thymus tissues were dissected and collected carefully. These tissues were washed with physiological saline, and then dried with filter paper. The lung index and inhibition ratio of lung index were calculated based on the following formulas as the markers for pulmonary edoema based on the previous research (Wan et al. 2014). The spleen index and thymus index were calculated based on the following formulas as indicator of immune function (Smetannikova et al. 2006). The formulas are as follows:

Lung index = (lung weight/body weight) × 100%;

Inhibition ratio of lung index = (the average lung index of virus control group − the average lung index of experimental group)/the average lung index of virus control group × 100%;

Spleen index = (spleen weight/body weight) × 100%;

Thymus index = (thymus weight/body weight) × 100%.

### Histological changes

On the third and fifth days after infection, six mice in each group were randomly euthanized by ether anaesthesia and bloodletting, and the lung tissues were collected. The right lung lobes of each mouse were washed with physiological saline and fixed in 10% formaldehyde solution for seven days. The fixed tissue samples were routinely processed, embedded in low-melting paraffin, sliced into sections with 3 ∼ 4 μm thickness and stained with haematoxylin and eosin (H&E), as previously described (Wei et al. 2019). Tissue lesions were observed under a double-blind examination using microscope.

### Real-time PCR (RT-PCR) analysis

On the fifth day after influenza virus infection, six mice in each group were randomly euthanized to collect lung tissues. The lung tissues were washed with saline, dried with filter paper, and then divided into two parts: the left lung lobe for RT-PCR analysis or western blot analysis, the remaining right lung lobes for the immunohistochemical analysis.

For RT-PCR analysis, the total RNA from the left lung lobes was extracted with RNeasy Mini Kit (QIAGEN, Germany), and finally dissolved in 40 μL of RNase-free water. Then RNA was reversed transcript to cDNA via PrimeScript^TM^ RT Master Mix (Takara, Dalian, China). The reaction conditions were 37 °C for 15 min, 85 °C for 5 s, 4 °C for 10 min. RT-PCR assay was performed on cDNA samples via the SYBR Premix Ex Tap^TM^ II (Takara, Dalian, China). The primer sequences which are used to amplify influenza virus M1 gene (IFV-M1), Bcl-2-associated X protein (Bax), B-cell lymphoma-2 (Bcl-2), cysteinyl aspartate specific proteinase 3 (caspase-3) and GAPDH (Table 1) were designed and synthesised by Sangon Biotech, Co., Ltd. (Shanghai, China). The reaction conditions were 95 °C for 2 min, followed by 95 °C for 15 s for 40 cycles of denaturation and extended at 55 °C for 35 s. RT-PCR analysis was used by the QuanStudio 12 K thermocycler (QuanStudio 12 K Flex Real-time PCR System, Applied Biosystems Co., USA). The relative expression levels of the target genes were quantified using the 2^-ΔΔCt^ method (Livak and Schmittgen 2001), and obtained by normalising to GAPDH.

**Table 1.** Primer sequences and product size used for real-time PCR analysis.

Gene	Product size(bp)	Forward(5’-3’)	Reverse(5′-3′)
M1	117	TCATTGGGATCTTGCACTTG	ACTTTGGCACTCCTTCCGTA
Bax	196	CCAGGATGCGTCCACCAA	AAAGTAGAAGAGGGCAACCAC
Bcl-2	184	CTACCGTCGTGACTTCGC	GGGTGACATCTCCCTGTT
caspase-3	225	ACAGCACCTGGTTACTATTC	CAGTTCTTTCGTGAGCAT
GAPDH	141	GCAAGTTCAACGGCACAG	CGCCAGTAGACTCCACGAC

### Immunohistochemistry analysis

The remaining right lung lobes were fixed with 10% formaldehyde solution and then soaked in paraffin subsequently. Paraffin-embedded lung tissue sections (3 ∼ 4 μm) were cut and processed as described previously. For the immunohistochemistry analysis, the 3 ∼ 4 μm lung tissue sections were incubated with anti-Bax (rabbit-anti-mouse polyclonal antibody, 1:100), anti-Bcl-2 (rabbit-anti-mouse polyclonal antibody, 1:100), anti-caspase-3 (rabbit-anti-mouse polyclonal antibody, 1:120) for 60 min at 37 °C, rewashed in PBS with 3 times, 5 min each time, and then used horseradish peroxidase coupled goat secondary antibodies (EnVision Two-Step kit, Denmark) incubated 40 min at 37 °C. The tissues were then in PBS washed 3 times for 5 min each time, and incubated in Diaminobenzidine (DAB) for 1 min. The lung tissue sections were finally counterstained with haematoxylin stain for 1-2 min, and dehydrated with step alcohol. Images were observed by an optical microscope (Leica Microsystems Ltd., Wetzlar, Germany) at 200× magnification. The positive cells in the nucleus or cytoplasm showed yellow or yellow-brown. In addition, the positive staining analysis of Bax, Bcl-2 and caspase-3 was performed according to the integrated optical density (IOD) of immunostaining in each field under five randomly selected fields by pathologists who were blinded to the different experimental conditions, using Image-ProPlus 6.0 software (Media Cybernetics, USA).

### Western blot analysis

The lung samples from the left lung lobe of each mouse were lysed in RIPA lysis buffer (Beyotime Biotechnology, Beijing, China) containing phenylmethanesulfonyl fluoride (PMSF) (Beyotime Biotechnology, Beijing, China) (Chen et al. 2015). The lysis solution was incubated on ice for 30 min, and then centrifuged at 12,000 *g* at 4 °C for 15 min to remove impurity. The protein concentration in the supernatants was determined by using the BCA protein assay kit (Beyotime Biotechnology, Beijing, China). The samples were denatured and separated by 15% SDS-PAGE and transferred to polyvinylidene difluoride (PVDF) membrane by electrophoretic transfer. Then they were incubated at 4 °C overnight with rabbit-anti-mouse polyclonal primary antibody of Bax (1:1000 dilution, Abcam ab32503, USA), Bcl-2 (1:500 dilution, Abcam ab692, USA) and caspase-3 (1:1000 dilution, Abcam ab13585, USA). Subsequently, the PVDF membranes were at 37 °C for 2 h incubated with the secondary horseradish peroxidase-conjugated anti-rabbit antibody of IgG. The blots were visualised with ECL-Plus reagent (GE Healthcare, Piscataway, NJ, USA). In these experiments, the gel images were performed with the Image J analysis software. Quantitative analysis of targeted bands was performed with β-actin to control the target protein loading.

### Statistical analysis

All experimental data were performed by one-way analysis of variance (ANOVA) using SPSS 20.0, followed by Tukey’s test, and displayed as the mean ± standard deviation (SD). The *P* value less than 0.05 was considered as statistically significant.

## Results

### Effects of YHPG on lung index, spleen index and thymus index

**Figure 1.**
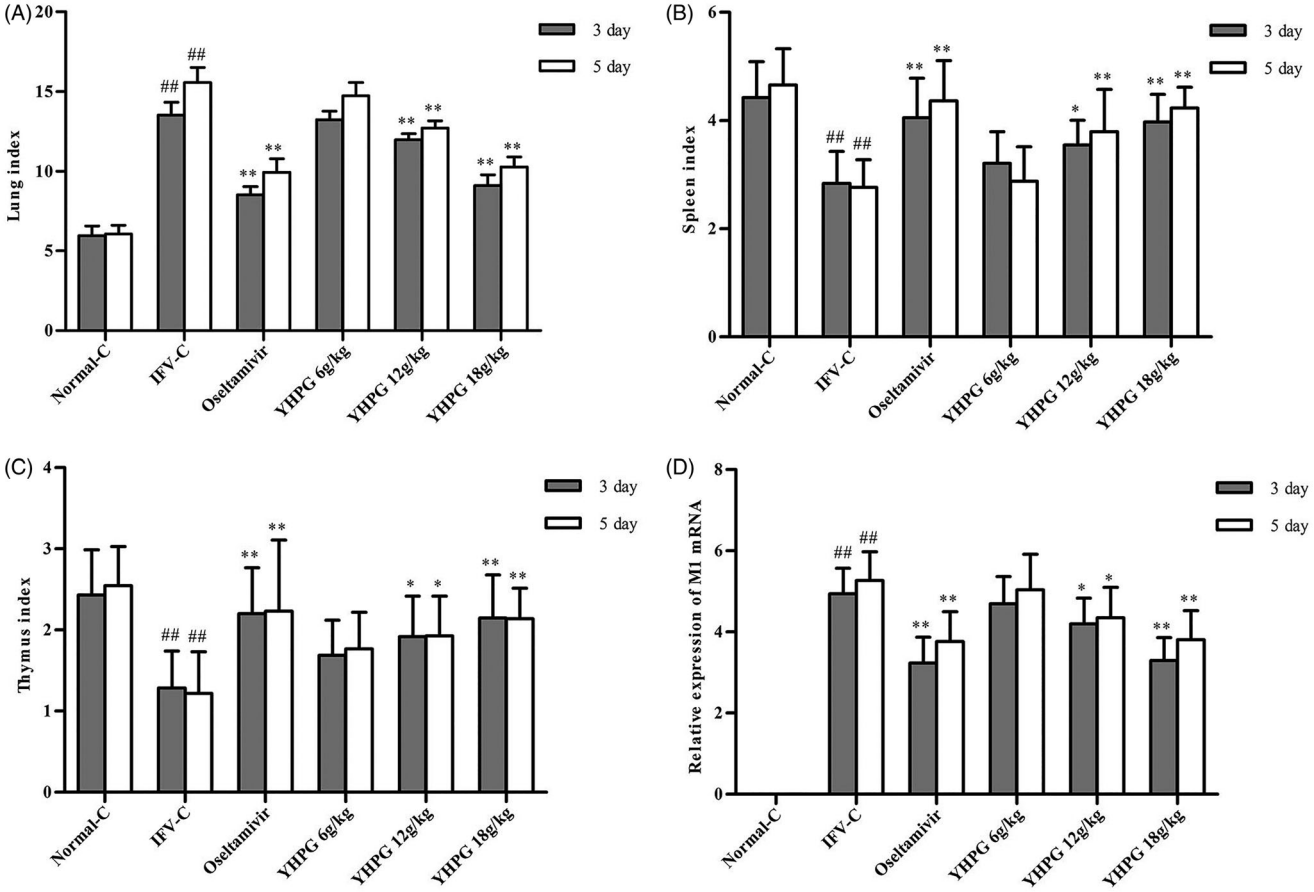
Effects of YHPG on the Lung (A), Spleen (B) and Thymus index (C) of IFV-infected mice. (D) The expression of viral loads in lung tissues of IFV-infected mice. ^##^*p* < 0.01 vs. Normal-C, *p < 0.05, **p < 0.01 vs. IFV-C.

The influenza virus caused viral pneumonia model in mice, and LD_50_ in mice determined by Reed-Muench method was calculated to be 10^−3.5^/0.02 mL. The lung index and inhibition ratio of lung index were used to evaluate pulmonary edoema. The spleen index and thymus index were used to evaluate immune function. As shown in Figure 1, after the third and fifth days of treatment, the lung index of YHPG (12 and 18 g/kg) or oseltamivir were significantly decreased compared with the IFV-C group (*p* < 0.01). The lung index of YHPG (6 g/kg) was also decreased, but there was no significant difference compared with the IFV-C group. And the inhibition ratio of lung index was detected at 2.19, 11.51, and 32.85% after the third day of treatment, 5.31, 18.22, and 34.06% after the fifth day of treatment at doses of 6, 12 and 18 g/kg YHPG, respectively (Table 2). After the third and fifth days of treatment, the spleen index and thymus index of YHPG (12 and 18 g/kg) or oseltamivir were significantly increased than those in the IFV-C group (*p* < 0.01 or *p* < 0.05). The spleen index and thymus index of YHPG (6 g/kg) was also increased, but there was no significant difference compared with the IFV-C group. These results showed that YHPG had dose-dependent antiviral effects in the ICR pneumonia mouse model by inhibiting pulmonary edoema and enhancing the immune organs function.

**Table 2.** The inhibition rate of lung index in IFV-infected mice treated with YHPG or oseltamivir.

Groups	Dose/g·kg^-1^	Inhibition rate of lung index/%	
		3 d	5 d
Normal-C	—	—	—
IFV-C	—	—	—
Oseltamivir	0.021	36.95	36.13
YHPG	6	2.19	5.31
YHPG	12	11.51	18.22
YHPG	18	32.85	34.06

### Effects of YHPG on viral loads in the lung tissues

To assess the effects of YHPG on viral replication, the viral load expression was calculated infected with influenza virus. As shown in Figure 1(D), no viral load expression was detected by real-time PCR in the Normal-C group, the viral load was significantly increased after influenza virus infection (*p* < 0.01), and the viral load on the fifth day after IFV-infected was higher than that on the third day. After the third and fifth days of treatment, the viral load in the YHPG (12 and 18 g/kg) or oseltamivir group was significantly lower than that in the IFV-C group (*p* < 0.01); the viral load of YHPG (6 g/kg) was also increased, but there was no significant difference compared with the IFV-C group. These results indicated that treatment of YHPG could inhibit influenza virus replication in the lung tissues of mice infected with influenza virus.

### Effects of YHPG on viral-induced lung pathological damage

**Figure 2.**
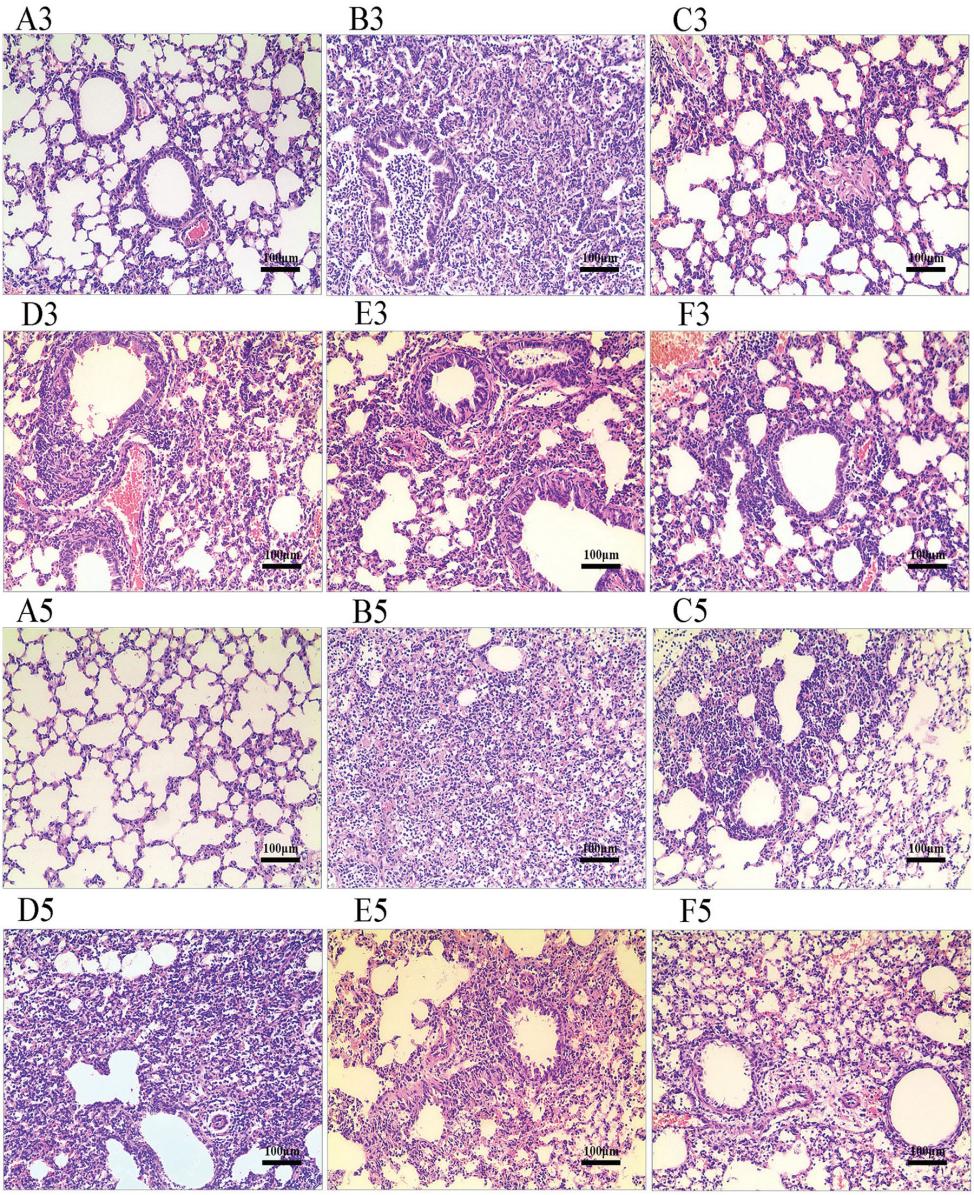
Effects of YHPG on viral-induced lung pathological changes. The lung tissues from each experimental group were calculated for histological evaluation: (**A**) Normal control group (Normal-C); (**B**) IFV control group (IFV-C); (**C**) IFV-infected and treated with oseltamivir (21.63 mg/kg/day); (**D**, **E** and **F**) IFV-infected and treated with 6, 12 and 18 mg/kg/day of YHPG, respectively. Representative histological section of the lung tissues from each group was stained by haematoxylin and eosin (magnification 200×). In the H&E staining microphotographs, the number 3 and 5 refer to the third and fifth day after IFV-infected, respectively.

We observed histopathological examination of lung tissues by an optical microscope to further analyse the effect of YHPG in mice infected with IFV. As shown in Figure 2, there were no obvious histological changes in the lung tissues of the Normal-C group. In contrast, lung tissues in the IFV-C group showed marked infiltration of macrophages and monocytes, necrosis of bronchial epithelial cells, extensive lung consolidation, interstitial edoema, haemorrhage, widespread thickening of alveolar wall, and destruction of alveolar structure. Additionally, more severe pathological damage was observed in murine lung tissues on the fifth day. After the third and fifth days of treatment, the IFV-induced lung pathological changes were alleviated in oseltamivir group and YHPG (6, 12, and 18 g/kg) group compared with the IFV-C group. These findings of the histological changes were consistent with lung index and lung viral load in lung tissues of mice infected with influenza virus. The results showed that YHPG could ameliorate lung tissue injury in IFV-infected mice.

### Effects of YHPG on the mRNA expression of Bax, Bcl-2 and caspase-3 by RT-PCR

**Figure 3.**
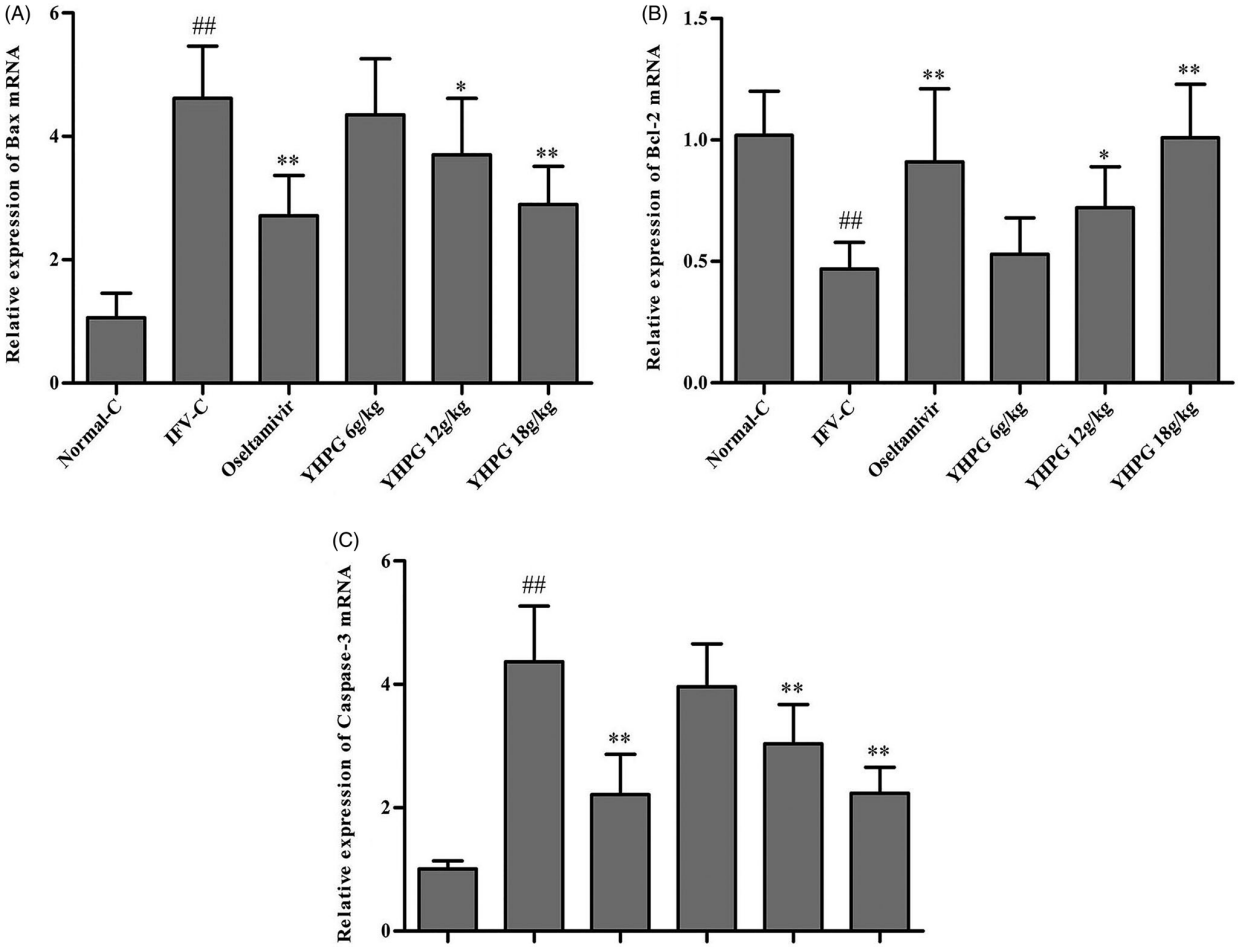
Effects of YHPG on the mRNA expression of Bax (A), Bcl-2 (B) and caspase-3 (C) in lung tissues of IFV-infected mice. ^##^*p* < 0.01 vs. Normal-C, *p < 0.05, **p < 0.01 vs. IFV-C.

The mRNA expression of Bax, Bcl-2 and caspase-3 in lung tissues was detected by RT-PCR, and these genes were the key targets of apoptosis signalling pathway. As shown in Figure 3, the results indicated that the mRNA expression of Bax and caspase-3 in lung tissues of IFV-C group were significantly up-regulated in comparison to the Normal-C group (*p* < 0.01), and the mRNA expression of Bcl-2 in lung tissues of IFV-infected group was significantly down-regulated compared with the Normal-C group (*p* < 0.01). Compared with IFV-C group, YHPG (12 and 18 g/kg) or oseltamivir significantly down-regulated the mRNA expression of Bax and caspase-3, and up-regulated the mRNA expression of Bcl-2 in lung tissues of mice infected with influenza virus on the fifth day (*p* < 0.01 or *p* < 0.05). However, there was no significant difference in mRNA expression of Bax, Bcl-2 and caspase-3 between the YHPG (6 g/kg) and IFV-C group. These results suggested that the YHPG could play dose-dependent antiviral effects in the ICR pneumonia mouse model by regulating the mRNA expression of the key targets Bax, Bcl-2 and caspase-3 in apoptosis signalling pathway in the lung tissues of mice infected with influenza virus.

### Effects of YHPG on the protein expression of Bax, Bcl-2 and caspase-3 by immunohistochemistry

**Figure 4.**
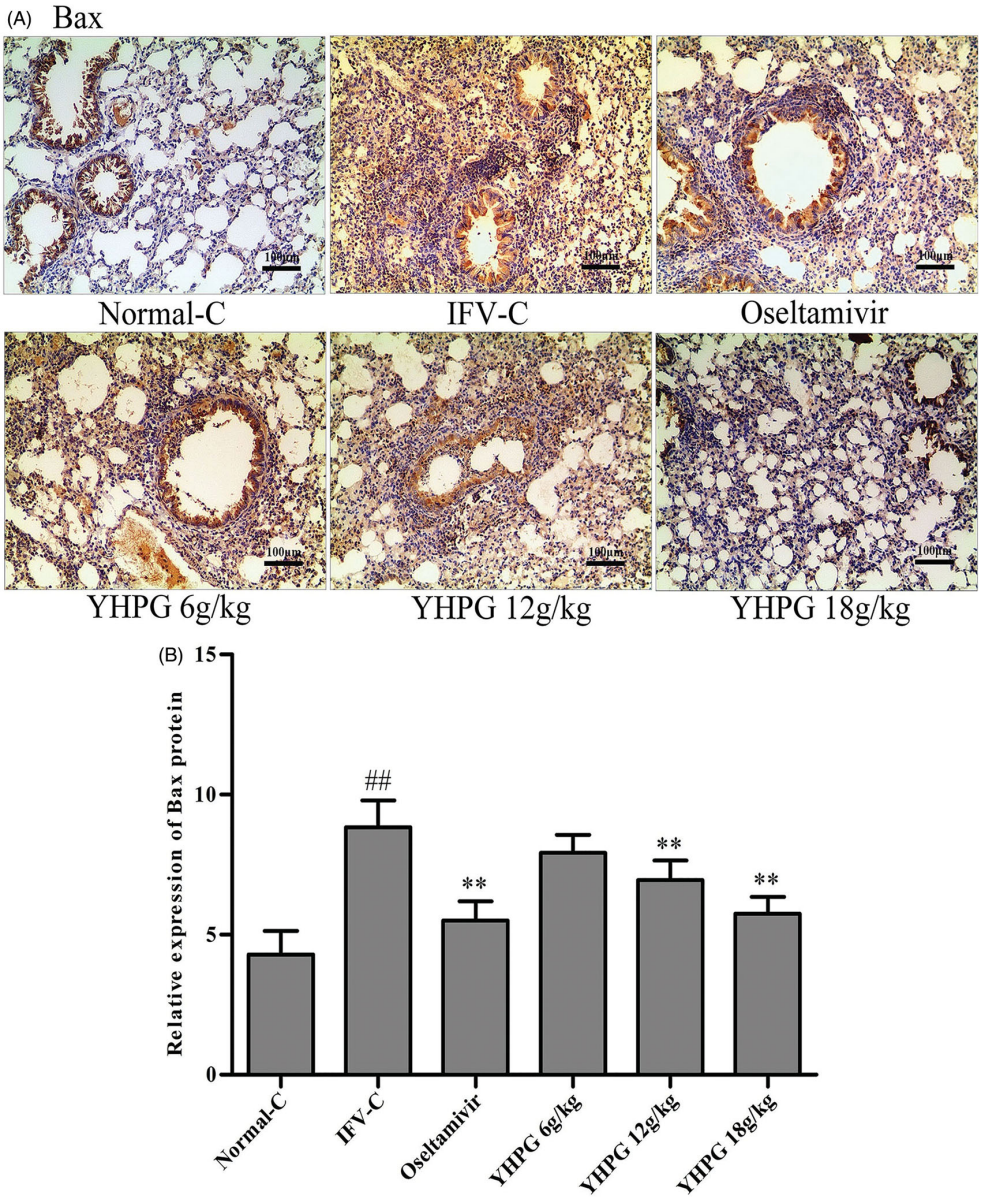
**A:** Immunostaining photomicrographs of Bax proteins in the lung tissues of IFV-infected mice (magnification 200×); **B:** Quantitative image analysis of Bax proteins was performed based on the integrated optical density (IOD) of positive immunostaining (brown) in the lung tissues from each group. ^##^*p* < 0.01 vs. Normal-C, *p < 0.05, **p < 0.01 vs. IFV-C.

**Figure 5.**
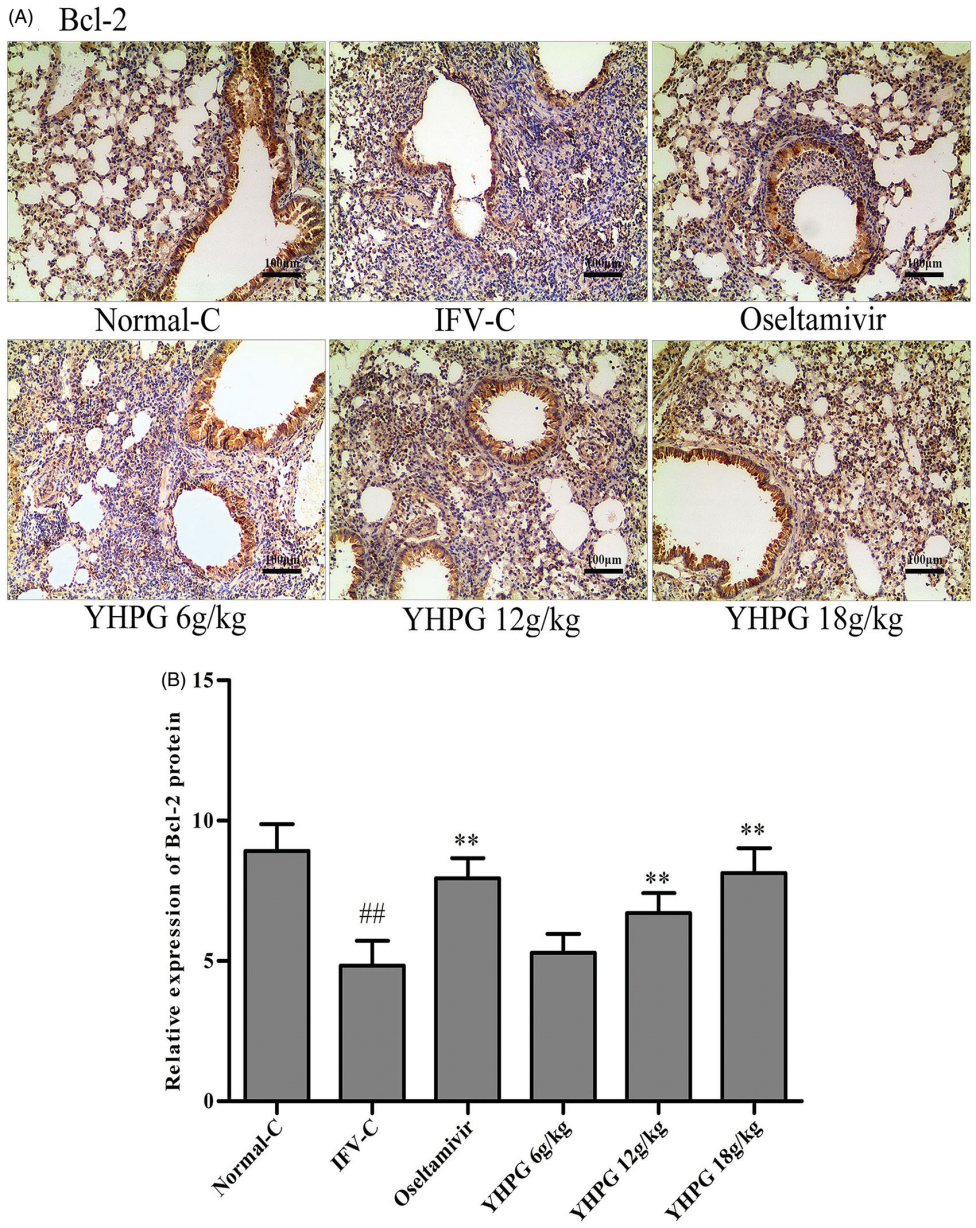
**A:** Immunostaining photomicrographs of Bcl-2 proteins in the lung tissues of IFV-infected mice (magnification 200×); **B:** Quantitative image analysis of Bcl-2 proteins was performed based on the integrated optical density (IOD) of positive immunostaining (brown) in the lung tissues from each group. ^##^*p* < 0.01 vs. Normal-C, *p < 0.05, **p < 0.01 vs. IFV-C.

**Figure 6.**
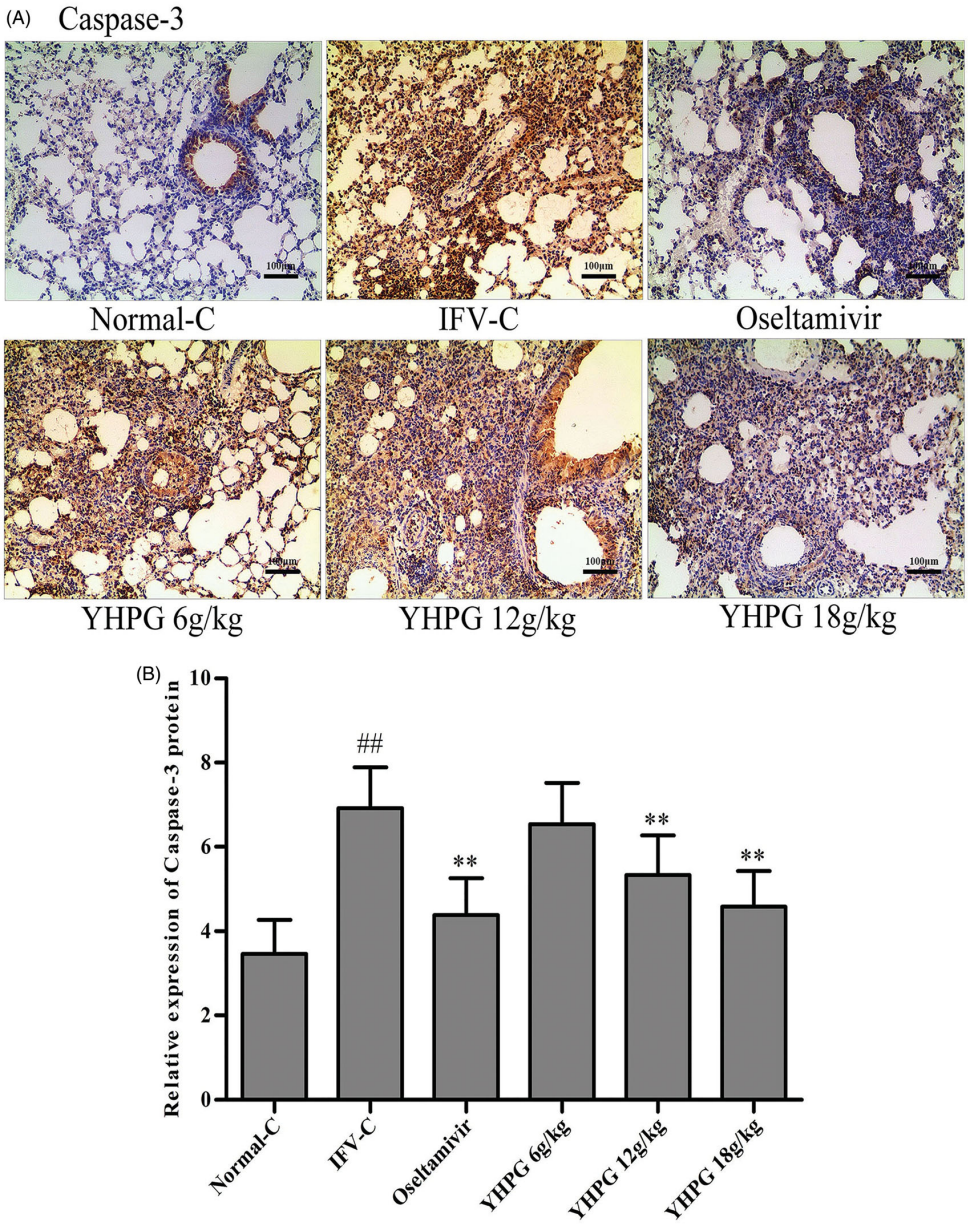
**A:** Immunostaining photomicrographs of caspase-3 proteins in the lung tissues of IFV-infected mice (magnification 200×); **B:** Quantitative image analysis of caspase-3 proteins was performed based on the integrated optical density (IOD) of positive immunostaining (brown) in the lung tissues from each group. ^##^*p* < 0.01 vs. Normal-C, *p < 0.05, **p < 0.01 vs. IFV-C.

The protein expression of Bax, Bcl-2 and caspase-3 as key targets of apoptosis signalling pathway was detected by immunohistochemistry staining. These photomicrographs in Figures 4–6 showed the protein expression of Bax, Bcl-2 and caspase-3 in the lung tissues, respectively. Among them, Bax and Bcl-2 positive cells are widely expressed in alveolar and airway epithelial cells, infiltrating inflammatory cells and vascular endothelial cells, and caspase-3 positive cells is mainly expressed in alveolar and airway epithelial cells. According to Figures 4–6, immunohistochemical assay found that the protein expression of Bax and caspase-3 were rarely detected in the lung tissues of Normal-C group mice, whereas their protein expression significantly increased in the IFV-C group (*p* < 0.01); while the protein expression of Bcl-2 were significantly decreased in the IFV-C group compared with the Normal-C group (*p* < 0.01). Compared with IFV-C group, YHPG (12 and 18 g/kg) or oseltamivir significantly decreased the protein expression of Bax and caspase-3, and increased the protein expression of Bcl-2 (*p* < 0.01); however, there was no significant difference in protein expression of Bax, Bcl-2 and caspase-3 between the YHPG (6 g/kg) and IFV-C group. We could summarise that YHPG regulated the protein expression of Bax, Bcl-2 and caspase-3 in the lung tissues of IFV-infected mice. These results were accordance with the mRNA expression of the key targets in apoptosis signalling pathway, as shown in Figure 3.

### Effects of YHPG on the protein expression of Bax, Bcl-2 and caspase-3 by Western blot

**Figure 7.**
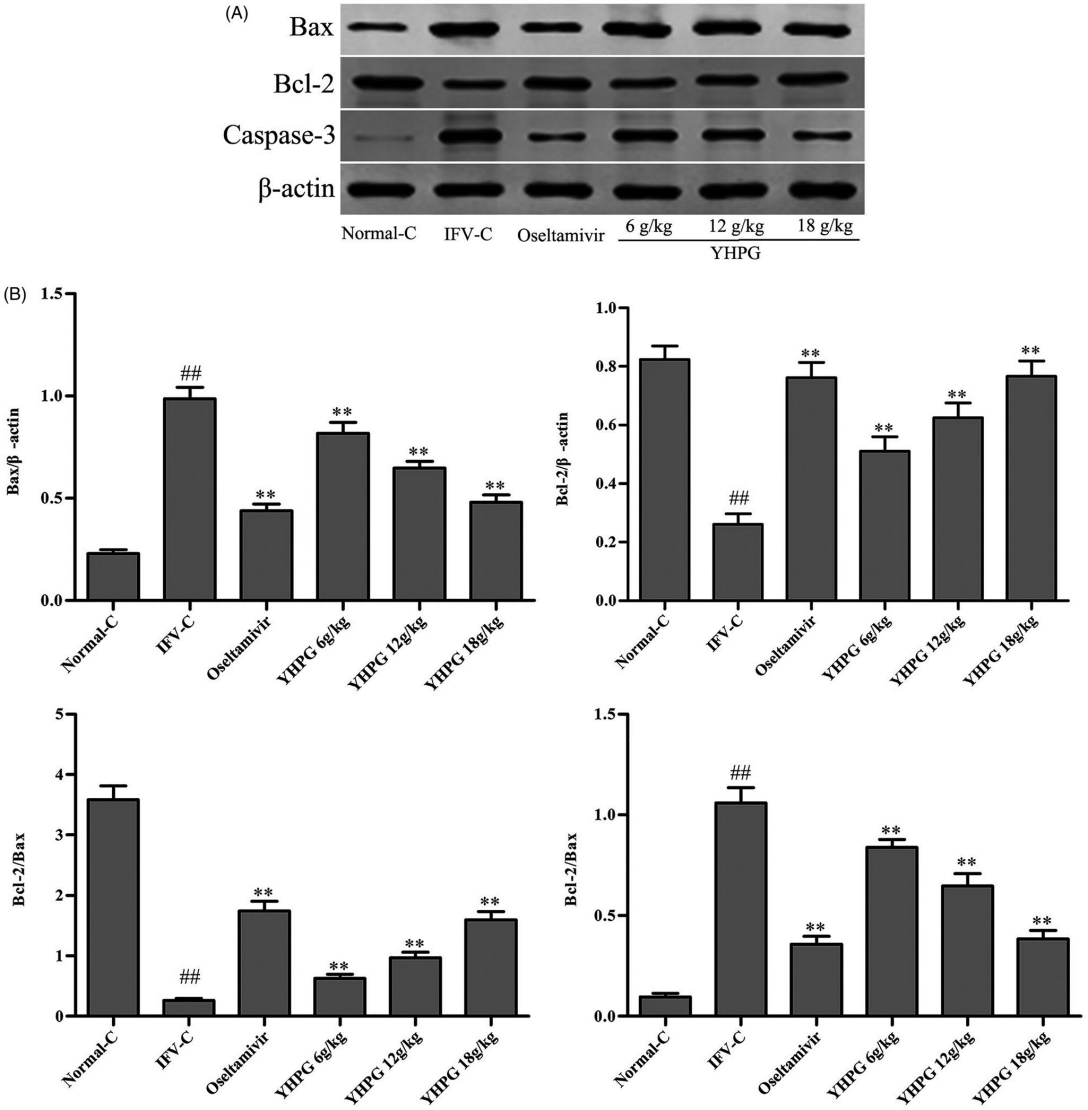
(A) Western blot showing the protein expression of Bax, Bcl-2 and caspase-3 in lung tissues of IFV-infected mice; (B) The quantitative analysis of Bax, Bcl-2 and caspase-3 expression, and the ratio of Bcl-2/Bax. ^##^*p* < 0.01 vs. Normal-C, *p < 0.05, **p < 0.01 vs. IFV-C.

The western blot analysis is commonly used to further investigate the protein expression of Bax, Bcl-2 and caspase-3. As shown in Figure 7, the results of the western blot assay showed that the protein expression of Bax and caspase-3 in the IFV-C group were significantly up-regulated (*p* < 0.01); while the protein expression of Bcl-2 and ratio of Bcl-2/Bax in the IFV-C group were significantly down-regulated in comparison to the Normal-C group (*p* < 0.01). By comparison with the IFV-C group, YHPG (6, 12 and 18 g/kg) or oseltamivir significantly ameliorated the protein expression of Bax and caspase-3; in the meantime, YHPG (6, 12 and 18 g/kg) or oseltamivir has higher levels of the protein expression of Bcl-2 and ratio of Bcl-2/Bax (*p* < 0.01). These results were consistent with the protein expression Bax, Bcl-2 and caspase-3 identified by immunohistochemical analysis.

## Discussion

Chinese herbal medicines are the most important component among the development of the traditional Chinese medicine system. Chinese herbal medicine has been used for the prevention and treatment of viral infectious diseases for thousands of years, especially in China and many other Asian countries clinical practice (Dan et al. 2013; Choi et al. 2016; Zhong et al. 2016). Compared with great majority of antiviral drugs consisting of a single component, Chinese herbal compounds with multiple components are considered to have multiple biological activities and multiple targets. Moreover, TCM has been widely accepted by some places in poor financial condition, especially in the treatment of chronic diseases (Zhang et al. 2012). At present, the traditional Chinese herbal compounds used for the respiratory viral infection account for the majority of antiviral drugs on the market. Oseltamivir carboxylate, widely used in the treatment of influenza virus A or B infection, has high bioavailability and penetrability and is sufficient to inhibit viral replication (Zhu & Markowitz 2009; Davies 2010). However, the emergence of oseltamivir resistance and the global spread of influenza viruses during the 2007-2009 emphasise the need for sustained surveillance of antiviral drug susceptibilities (Samson et al. 2013). WHO recommends the use of oseltamivir as an antiviral western medicine against global influenza virus pandemic’ outbreaks. In order to scientifically estimate the antiviral effects of YHPG, oseltamivir was used for a positive control in this study.

As a modified Chinese herbal compound, YHPG has a variety of pharmacological effects, with the advantages of high efficiency, low toxicity, convenient utilisation and quality control. Previous researches have shown that YHPG has distinct antitussive, anti-inflammatory and antibacterial effects (He et al. 2014; Wan et al. 2016). Further, antiviral studies has demonstrated that YHPG has significant effects on ameliorating influenza virus-induced inflammation, inhibiting TLRs/MyD88 mediated NF-κB signalling pathway, and inhibiting influenza virus replication (Peng et al. 2016a, 2016b). However, the antiviral effects and its possible mechanism of YHPG in the apoptosis signalling pathway are still obscure. In this experiment, we performed the antiviral effects of YHPG, then selected the key targets Bax, Bcl-2 and caspase-3 in the apoptosis signalling pathway to explore its possible mechanism on mice with influenza viral pneumonia.

Antiviral studies showed that the model of influenza viral pneumonia was successfully established in mice, and YHPG (12 and 18 g/kg) significantly decreased lung index and viral load in the IFV-infected lung tissues, increased spleen index and thymus index, and remarkably ameliorated lung pathological lesions in the IFV-infected group. As mentioned in the methods, a large number of studies have shown that lung index plays an important role in indicating the severity of lung tissue pathological damage in mice with influenza viral pneumonia (Deng et al. 2013). After the influenza virus invades the body, it will cause damage to immune organs, which may lead to the occurrence of apoptosis (Wong 2011). The spleen index and thymus index can be used as indicators of immune function to reflect the immune status to some extent. Pathological analysis revealed that inflammation and consolidation of IFV-infected mice increased significantly, which was consistent with our research (Qu et al. 2016). In addition, we also found that YHPG could inhibit influenza virus replication, reduce the expression of M1 gene, and M1 gene indicate the synthesis of influenza viral gene (Choi et al. 2017). Therefore, it can be concluded that YHPG has significant antiviral effects in mice infected with influenza virus by regulating the lung index, spleen index and thymus index, inhibiting influenza virus replication, and alleviating lung pathological lesions.

Antiviral test results showed that the lung index and viral load on the fifth day after infection with IFV were higher than those on the third day; in addition, more severe pathological damage was observed in murine lung tissues on the fifth day. Therefore, we chose the fifth day after infection to investigate its possible mechanism focus to the apoptosis signalling pathway.

Induction of cell apoptosis is a complex multi-factor reaction in the host defense and pathogenesis of IFV, and different influenza virus strains have different ability to induce cell apoptosis (Liu et al. 2014). As we know, apoptosis is an initiative process, which involves a series of functional significance associated with cell proliferation, influenza virus transmission, inherent host defense, and lung tissue damage during the viral replication cycle such as genes activation, expression and regulation (Herold et al. 2012; Pei et al. 2014). Apoptosis also plays an important role in the pathogenesis of many diseases, and too much apoptosis may lead to degenerative diseases and too little apoptosis may result in malignant cell death; moreover, defects may occur at any point in the apoptosis signalling pathway (Wong 2011). Influenza virus usually causes caspase-dependent apoptosis activated by caspase-3, leading to nuclear export of newly synthesised viral nuclear protein (NP) and increased influenza virus replication (Takahashi et al. 2013); in addition, antiviral drugs may dampen apoptosis of H1N1-infected cells by reducing the functional activation of caspase-3 (Zhang et al. 2017). Furthermore, the mitochondrial pathway is the main pathway of apoptotic cell death, mainly controlled by Bcl-2 family proteins, and the family members mainly include Bcl-2 and Bax, which have both anti-apoptotic and pro-apoptotic effects (Dewson and Kluck 2010). And the ratio of Bcl-2/Bax is considered to be a key indicator for estimating homeostasis states and mitochondrial membrane potential in cell survival (Malla et al. 2010). To further understanding the antiviral effects of YHPG on lung epithelial cells of IFV-infected mice through inhibition of apoptosis, we used real-time PCR, immunohistochemical analysis and Western blot assays, which belonged to semi-quantitative, quantitative and qualitative analysis, to evaluate the mRNA and protein expression of Bax, Bcl-2, and caspase-3, which are the key targets in apoptosis signalling pathway (Puerto et al. 2010).

In our results, the expression of Bax and caspase-3 in the IFV-C group was significantly up-regulated in comparison with the Normal-C group, while the expression of Bcl-2 and the ratio of Bcl-2/Bax in the IFV-C group were reversely down-regulated, suggesting that apoptosis of mouse lung tissues occur after influenza virus infection, which may through the mitochondrial pathway dominated by Bcl-2 family and caspase-dependent apoptosis pathway activated by caspase-3. Compared with influenza virus infection control group, YHPG (12 and 18 g/kg) or oseltamivir significantly down-regulated the expression of Bax and caspase-3, and significantly up-regulated the expression of Bcl-2 and the ratio of Bcl-2/Bax. That suggests that YHPG could protect lung tissue of IFV-infected mice from apoptosis, and its possible antiviral mechanism might be associated with regulation of the expression of Bax, Bcl-2, and caspase-3 in the key targets of the apoptosis signalling pathway.

However, the present study has some limitations. We did not investigate the time course of influenza virus growth-inhibition and apoptosis of YHPG, which is a better indication of the potential mechanism of YHPG against apoptosis caused by influenza virus infection observed in this study.

## Conclusions

Our data revealed that YHPG has significant antiviral effects in mice with influenza viral pneumonia, which might be related to the inhibition of influenza virus replication, alleviation of lung tissue damage, protection of immune organs and regulation of the expression of Bax, Bcl-2, and caspase-3 in the key targets of apoptosis signalling pathway. Although the exact antiviral mechanism of YHPG through inhibition of apoptosis remains to be further explored, our results suggest that YHPG exerts antiviral effects via regulating Bax, Bcl-2, and caspase-3 associated with apoptosis in mice with influenza viral pneumonia, which may provide an effective and alternative approach for therapy of influenza virus infection in clinical trials.

## Supplementary Material

Supplementary_Table_1.docx

Supplementary_Figure_1.jpg

## Data Availability

The relevant datasets used to support the findings of this manuscript are available from the corresponding author by reasonable request.
