# Supplementary material for: Preliminary study of Yinhuapinggan granule against H1N1 influenza virus infection in mice through inhibition of apoptosis
Source: Pharm Biol. 2020 Sep 23;58(1):979–91. doi: 10.1080/13880209.2020.1818792 (PMC7534346; doi:10.1080/13880209.2020.1818792)
Supplement: Supplementary_Table_1.docx [file IPHB_A_1818792_SM8831.docx]

Supplementary Table 1 Component herbs of YHPG

| Pharmaceutical name | Botanical plant name | Family | Weight (g) | Used part |
| --- | --- | --- | --- | --- |
| Radix Puerariae Lobatae | *Pueraria lobata* (Willd.) Ohwi | Lamiaceae | 10 | Radix |
| Flos Lonicerae Japonicae | *Lonicera japonica* Thunb. | Caprifoliaceae | 10 | Flower bud |
| Polygoni Cuspidati Rhizoma | *Polygonum cuspidatum* Sieb.et Zucc. | Polygonaceae | 10 | Root and rhizome |
| Ephedrae Herba | *Ephedra sinica* Stapf. | Ephedraceae | 5 | Aerial part |
| Armeniacae Semen Amarum | *Prunus armeniaca* L. | Rosaceae | 5 | Fruit |
| Glycyrrhizae Radix | *Glycyrrhiza uralensis* Fisch. ex DC*.* | Leguminosae | 2.5 | Root and stolon |
