# Supplementary figures and images for: Preliminary study of Yinhuapinggan granule against H1N1 influenza virus infection in mice through inhibition of apoptosis
Source: Pharm Biol. 2020 Sep 23;58(1):979–91. doi: 10.1080/13880209.2020.1818792 (PMC7534346; doi:10.1080/13880209.2020.1818792)

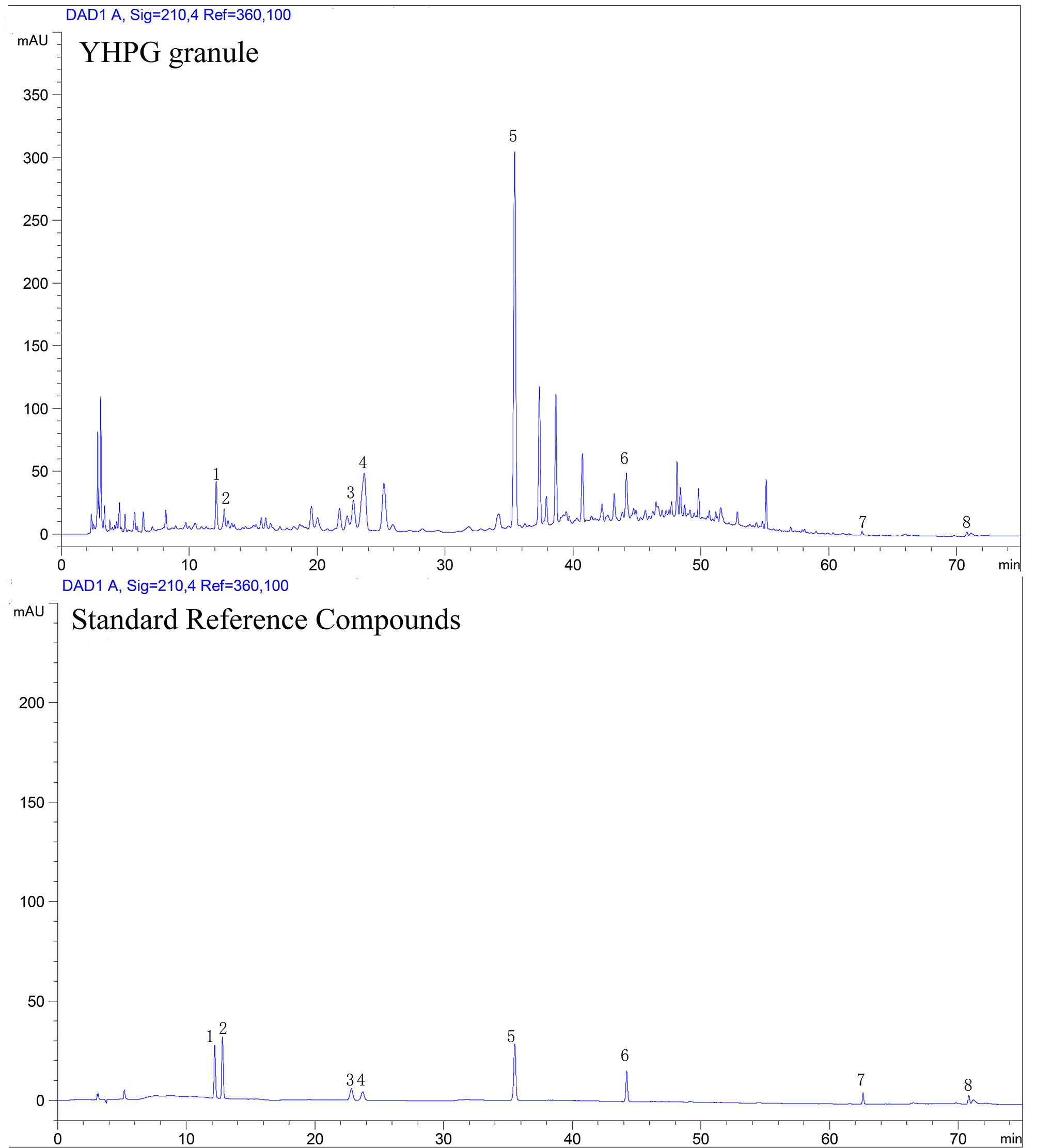

Supplement: Supplementary_Figure_1.jpg [file IPHB_A_1818792_SM8830.jpg]
